# Research on High-Precision Measurement Method for Small-Size Gears with Small-Modulus

**DOI:** 10.3390/s24165413

**Published:** 2024-08-21

**Authors:** Peng Niu, Qiang Cheng, Xinlei Zhang, Zhifeng Liu, Yongsheng Zhao, Congbin Yang

**Affiliations:** 1 Institute of Advanced Manufacturing and Intelligent Technology, Beijing University of Technology, Beijing 100124, China; niupeng100@163.com (P.N.); chengqiang@bjut.edu.cn (Q.C.); zhangxinlei@emails.bjut.edu.cn (X.Z.); yszhao@bjut.edu.cn (Y.Z.); 2 Jilin Provincial Key Laboratory of Advanced Manufacturing and Intelligent Technology for High-End CNC Equipment, Jilin University, Changchun 130025, China; lzfjlu@jlu.edu.cn

**Keywords:** small-modulus gears, gear measurement, 3D point cloud data, error model, workpiece coordinate system

## Abstract

Small-modulus gears, which are essential for motion transmission in precision instruments, present a measurement challenge due to their minuscule gear gaps. A high-precision measurement method under the influence of positioning errors is proposed, enabling precise evaluation of the machining quality of small-modulus gears. Firstly, a compound measurement platform for small-modulus gears is developed. Using a 3D model of the measurement system, the mathematical relationships governing motion transmission between various components are analyzed. Secondly, the formation mechanism of gear positioning error is revealed and its important influence on measurement accuracy is discussed. An optimization method for spatial coordinate transformation matrices under positioning errors of gears is proposed. Thirdly, the study focuses on small-sized gears with a modulus of 0.1 mm and a six-level accuracy. Based on the aforementioned measurement system, the tooth profile measurement points are collected in the actual workpiece coordinate system. Then, gear error parameters are extracted based on the established models for tooth profile deviation and pitch deviation. Finally, the accuracy and effectiveness of the proposed measurement method are verified by comparing the measurement results of the P26 gear measuring center.

## 1. Introduction

Small-modulus gears, which are a crucial precision drive component that finds extensive applications in aerospace, instrumentation, and intelligent manufacturing, among others [1,2], have various benefits, such as compact size, efficient transmission, and smooth operation. In addition, small-modulus gears need to satisfy high-precision, reliability, and load capacity requirements as related applications continue to improve. Errors in gear processing are becoming less common as manufacturing techniques get better and better. Therefore, precise measurement of small-modulus gears is essential to accurately evaluate gear quality [3].

As a critical component in motion transmission, the quality of small-modulus gears has a direct effect on the motion accuracy, noise, and lifespan of the instrumentation. Therefore, studying the measurement methods and error modeling of small-modulus gears to achieve high-precision measurements is a key technology problem to ensure the quality of the instrument [4,5]. The significance of micro-drive systems will become increasingly more and more obvious due to the enormous demand for gears in related application sectors. However, the size of gears is becoming smaller and smaller, and the precision level is rising. The development of micro gears in particular creates new challenges for small-modulus gears of measurement.

The two main categories of measurement techniques for small-modulus gears are contact measurement and non-contact measurement. Contact measurement generally uses a coordinate measuring machine (CMM) with a contact probe, a double-flank meshing measurement instrument, and other instruments to collect measurement points for the gear evaluation section [6,7,8]. For small-modulus gears, the contact probe is challenging to insert into the tooth profile, and its rigidity is inadequate. The contact force during the measurement will cause the gear to deform, which will reduce the precision of the measurement. Currently, the contact measuring instruments that are commonly used are unable to satisfy the measurement requirements of small-modulus gears. Therefore, optical measurement, which improves measurement efficiency and is extensively used, is the main focus of research in small-modulus gear measurement [9].

Guo et al. [10] built a 3D point cloud measurement system for gears based on a line-structured light sensor and acquired millions of 3D data points on the gear tooth flank in 5 s. Miao et al. [11] investigated the vision measurement of radial runout of gear shafts based on line-structured light, which is fast and does not require the gear shaft to be rotated. Shang et al. [12] investigated a high-precision contour measurement method using non-coherent line-structured light, and the tooth shape error of the measured involute template was ±2.2 μm, which was higher than that of the line laser method. Urbas et al. [13] investigated a gear measurement method based on 3D optical metrology, performed measurement experiments on gears, and compared them with CMM gear measurement experiments to verify the practicality of the method. Chen et al. [14] established a non-contact optical inspection system based on the projected trajectory method with a system accuracy of 2.76 μm and a mean value of 3 μm for the measured tooth profile deviation. Luo et al. [15] investigated the optical coherence chromatography-based measurement method for small-modulus gears and verified the feasibility of the method by measuring 0.2 mm and 0.5 mm modulus gears, and the results show that the measurement resolution is less than 10 μm, which could be used to measure gears with modulus less than 0.2 mm. Kumar et al. [16] proposed pre-processing with a k-means averaging algorithm to improve the measurement accuracy of laser sensor measurements after obtaining data with 3D laser measurements but did not evaluate the gear accuracy. Shi et al. [17] proposed a method for rapid measurement of gear pitch using a point laser sensor, which can complete the measurement of gears according to different measurement efficiency and accuracy requirements. Fang et al. [18,19] studied the laser interference gear measurement method based on a CCD image sensor. The measurement objects are involute spur gear and helical gear, and a systematic improvement scheme is proposed. However, the aforementioned optical measurement techniques for measuring gears have strict requirements for the angle of incidence and reflection. Unreasonable angle positions will occlude the tooth flank in this area, or the secondary reflected light generated in this area will enter the sensor receiving area, which will influence the measurement results, especially for the sections of tooth root and tooth tip with large curvature.

For the above problems, a small-modulus gear measurement system of high-precision composite coordinate measuring machine with an optical fiber sensing device is established. The optical-based “contact-optical” measurement method can achieve high-precision measurements of small-modulus gears with 0.1 mm modulus 6-grade precision.

## 2. Measurement System of Small-Modulus Gears

**Figure 1:**
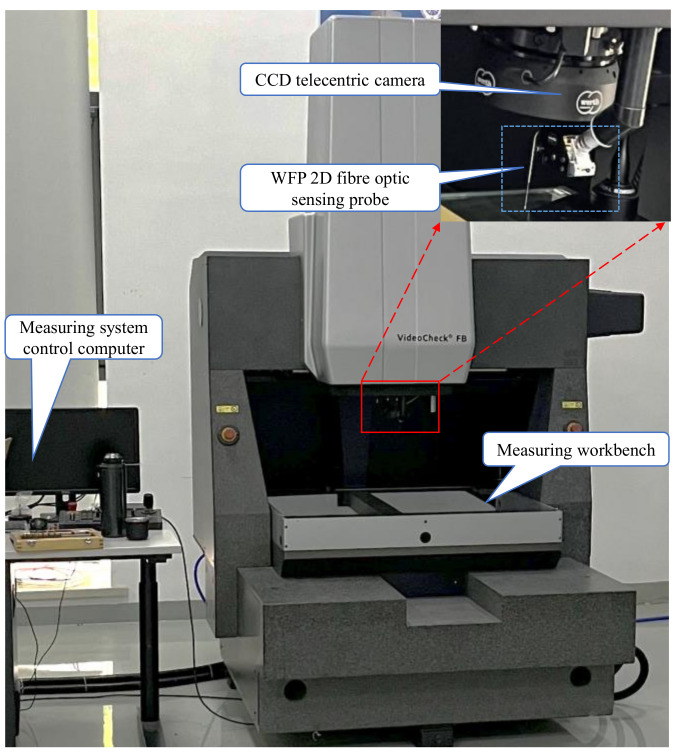
Small-modulus gears measurement platform.

In this paper, a fiber optic sensing device (WFP) and composite CMM are used to establish the gear measurement platform, as shown in Figure 1. CMM employs a three-axis linkage to convert the motion relationship, whose parameters can be found in Table 1. The fiber-optic sensing device mainly consists of a light source, fiber optic measurement unit, CCD telecentric camera, and image processing unit, among others. Table 2 presents its principal parameters. The CCD telecentric camera is highly advantageous in terms of precise imaging and strong anti-interference ability [20,21]. It can accurately capture the position of the self-luminous detection element optically and measure the offset of the micro-detection element when the measured workpiece moves. In the movement of the workpiece, the accurate position of the micro-detection element is superimposed with the coordinates of the equipment calibration system to extract the measurement points.

Before taking measurements, it is necessary to adjust the optical fiber sensor to the plane of the object within the optical system. Following the provision of the light source, a bright spot is created on the CCD image sensor through the objective lens by the light reflected or diffusely reflected by the optical fiber probe. In this method, the contact force between the probe and the small-modulus gear is very small during the measurement, which avoids the deformation or fracture of the gear or probe due to the measurement force. It is not necessary to consider the influence of micro-contact force on the measurement results [22].

### 2.1. Modeling of Mathematical Motion Relation

**Figure 2:**
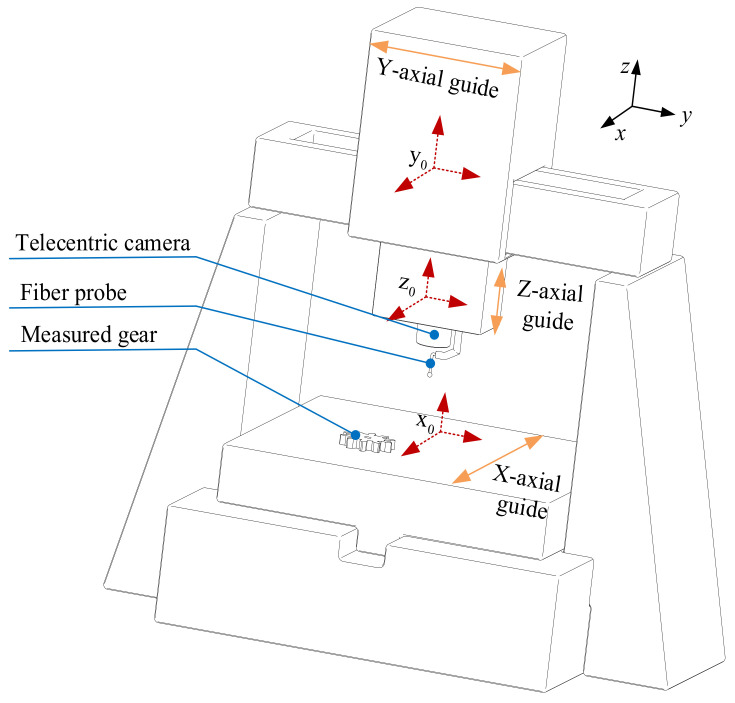
3D modeling of measuring system of small-modulus gears.

To ensure measurement accuracy when measuring small-modulus gears, it is essential to establish the relative position relationship between the gears and the fiber optic sensor. Therefore, in this paper, a three-dimensional model of the measurement system is established based on a measurement platform to study the mathematical motion relationship between each component, as shown in Figure 2.

According to the three-dimensional model of the system, there are three coordinate systems in the measurement system, which are the machine coordinate system, workpiece coordinate system, and CCD telecentric camera coordinate system. The linear motion of the guide rails for the X, Y, and Z axes can be considered as linear motion along the three coordinate axes, resulting in the establishment of a fixed coordinate system for the linear motion of the three axes. To obtain the three-dimensional point cloud data of the tooth flank on the measured workpiece coordinate system, it is necessary to pass through six-coordinate systems. The six-coordinate systems include CCD telecentric camera, Z-axis, Y-axis, X-axis, machine, and the workpiece coordinate systems.

Under ideal conditions, based on the coordinate transformation matrix of the measurement system, the transformation parameters between the coordinate systems can be determined to realize the transfer of coordinate data. However, it is necessary to consider the actual factors between the coordinate systems of the measurement system to optimize the coordinate transfer matrix for improving the accuracy of coordinate extraction.

### 2.2. Coordinate Transformation Model

**Figure 3:**
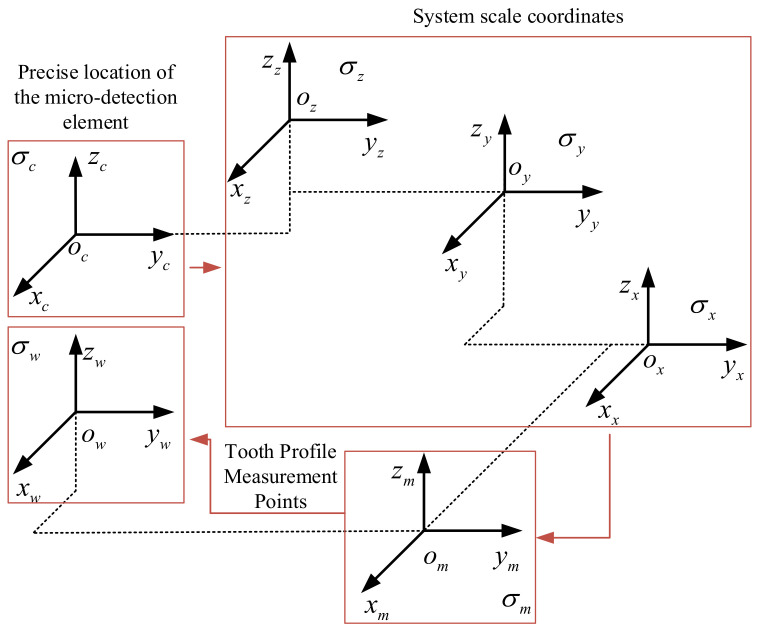
The position and geometric relationship of the coordinate system.

As shown in Figure 3, under ideal conditions, based on the three-dimensional model of the measurement platform and the motion relationship of the three axes, the coordinate transformation model of the measurement system is established, and the spatial coordinate transformation matrix of the whole system is derived to realize the transformation of the 3D point cloud data for small-modulus gears to the workpiece coordinate system.

Let the general coordinate transformation matrix between spatial coordinate systems be:

(1) $$T_{j}^{i}=\left(\begin{array}{cc} A_{j}^{i} & D_{j}^{i}\\ 0 & 1 \end{array}\right)$$

where $T_{j}^{i}$ represents the transformation matrix from coordinate system i to coordinate system j. The matrix represents the 3×3 transformation matrix of angular displacement between two adjacent coordinate systems, Vector represents the 3×1 vector parameter of linear displacement transformation between two adjacent coordinate systems, which can be expressed as:

(2) $$A_{j}^{i}=\left\{\begin{array}{l} E\;\;\;i,j\in \{c,z,y,x,m\},i\neq j\\ \left(\begin{array}{ccc} a & b & c\\ e & f & h\\ h & i & j \end{array}\right)\;i\;or\;j\in w,i\neq j \end{array}\right.$$

(3) $$D_{j}^{i}={\left(-x_{ji},-y_{ji},-z_{ji}\right)}^{T}$$

where E represents the 3×3 unit matrix; a→j represent the angular displacement transformation parameters. $x_{ji}\to z_{ji}$ represent the linear displacement transformation parameter.

The data of the three-dimensional point data of the small-modulus gear tooth profile in the ideal workpiece coordinate system $o_{w}-x_{w}y_{w}z_{w}$ are expressed as $P_{w}={\left(x_{w},y_{w},z_{w}\right)}^{T}$ and the exact positional coordinates of the micro-detection element in the CCD telecentric camera coordinate system are expressed as $P_{c}={\left(x_{c},y_{c},z_{c}\right)}^{T}$. Based on the general coordinate transformation matrix, the tooth profile measurement points satisfy:

(4) $$\left(\begin{array}{c} P_{w}\\ 1 \end{array}\right)=T_{f}^{w}\cdot \left(\begin{array}{c} P_{c}\\ 1 \end{array}\right)$$

Based on Equation (1), $T_{w}^{m}$ can be expressed as:

(5) $$T_{f}^{w}=T_{m}^{w}\cdot T_{x}^{m}\cdot T_{y}^{x}\cdot T_{z}^{y}\cdot T_{c}^{z}$$

**Figure 4:**
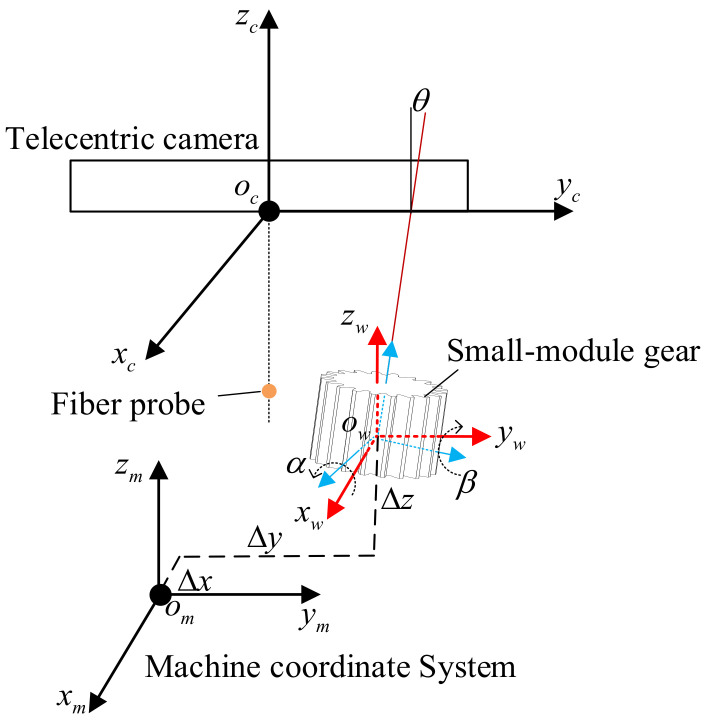
Model of gear positioning error.

Due to the positioning error of gears, the gear cross-section can become tilted [23]; there will be a discrepancy between the collected point cloud data of the gear tooth profile and the theoretical model, which can affect the evaluation of quality and performance of the small-modulus gears. It is required to optimize the coordinate transfer matrix of the measurement system to eliminate the impact of positioning errors and improve the measurement precision of gears. Therefore, this paper proposes a method to correct the positioning error of small-modulus gears. The proposed method optimizes the coordinate transfer matrix by establishing the positioning error model of gears, as shown in Figure 4.

**Figure 5:**
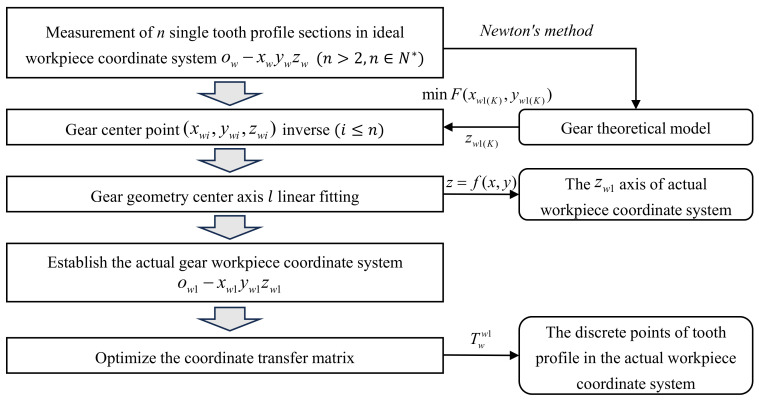
Positioning error correction flow chart.

The specific process is shown in Figure 5. In this study, a small-modulus gear with a modulus of 0.1 mm is used for the experiments. It is necessary to measure the point cloud data of a single tooth profile section in the ideal workpiece coordinate system. Due to the small size of the gear, the measured tooth profile containing the positioning error can be regarded as a standard tooth profile to determine the center point of the single tooth profile section.

**Figure 6:**
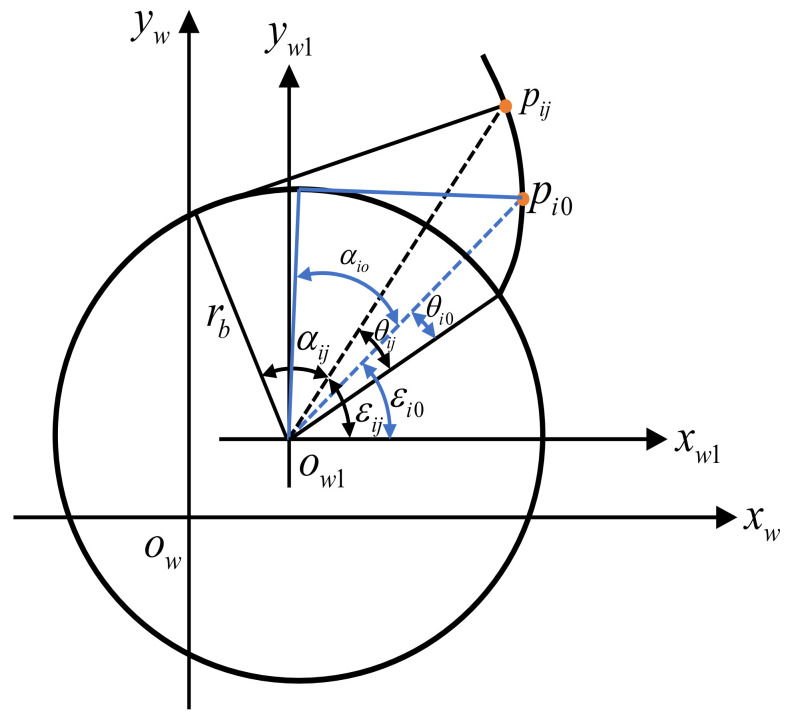
The center point of the single tooth profile section.

Under actual conditions, there are machining errors in the machining process of gears. As shown in Figure 6, in the ideal workpiece coordinate system located in the two-dimensional plane, the least squares can be established according to the geometric relationship between any two points *p_ij_* (*x_ij_*,*y_ij_*) and *p_i_*_0_ (*x_i_*_0_,*y_i_*_0_) on the gear involute, which can be expressed as:

(6) $$F(x_{w1},y_{w1})=\min\sum_{i=1}^{M}\sum_{j=1}^{N}(\Delta_{ij}{)}^{2}$$

(7) $$\Delta_{ij}=\Delta \theta_{ij}-\Delta \varepsilon_{ij},\Delta \theta_{ij}=\theta_{ij}-\theta_{i0},\Delta \varepsilon_{ij}=\varepsilon_{ij}-\varepsilon_{i0}$$

where *i* represents the number of involutes. *j* represents the number of measurement points on the *i*-th right involute line. $\theta_{ij}$ and $\theta_{i0}$ represent the spreading angles corresponding to points $p_{ij}$ and $p_{i0}$, respectively. $\varepsilon_{ij}$ and $\varepsilon_{i0}$ represent the angle of inclination corresponding to the lines $o_{w1}p_{ij}$, and $o_{w1}p_{i0}$, respectively.

Based on the involute theory of cylindrical gears, there are:

(8) $$\begin{array}{l} \Delta_{ij}=\frac{\sqrt{{\left(x_{ij}-x_{w1}\right)}^{2}+{\left(y_{ij}-y_{w1}\right)}^{2}-r_{b}^{2}}}{r_{b}}-\arccos\frac{r_{b}}{\sqrt{{\left(x_{ij}-x_{w1}\right)}^{2}+{\left(y_{ij}-y_{w1}\right)}^{2}}}\\ -\left(\frac{\sqrt{{\left(x_{i0}-x_{w1}\right)}^{2}+{\left(y_{i0}-y_{w1}\right)}^{2}-r_{b}^{2}}}{r_{b}}-\arccos\frac{r_{b}}{\sqrt{{\left(x_{i0}-x_{w1}\right)}^{2}+{\left(y_{i0}-y_{w1}\right)}^{2}}}\right)\\ -\left(\arctan\frac{y_{ij}-y_{w1}}{x_{ij}-x_{w1}}-\arctan\frac{y_{i0}-y_{w1}}{x_{i0}-x_{w1}}\right) \end{array}$$

Based on Newton’s iterative algorithm for solving the optimal solution of Equation (6), the iteration condition for iterating Equation (9) is $\left\|\nabla F(x_{w1(k+1)},y_{w1(k+1)}\right\|\leq \psi$.

(9) $${\left[x_{w1(k+1)},y_{w1(k+1)}\right]}^{T}={\left[x_{w1(k)},y_{w1(k)}\right]}^{T}-\frac{1}{H_{\left(k\right)}}\cdot \nabla F_{\left(k\right)}$$

where *k* represents iteration times, ∇F represents the gradient of Equation (6), and H represents the Hessian matrix of Equation (6).

Let the coordinates of the center of a single section $z_{w1}=\sum_{i=1}^{z}\sum_{j=1}^{n}z_{ij}/\sum_{i=1}^{z}n_{i}$, $n_{i}$ represent the total number of items of point cloud data of a single tooth profile section.

Based on the least square method, the space line is linearly fitted according to the center coordinates (*x*_w1_, *y*_w1_, *z*_w1_) of different tooth profile sections in the ideal workpiece coordinate system. The fitting result is given by Equation (10) as the *z_w_*_1_ axis in the actual workpiece coordinate system.

(10) $$\frac{x-x_{0}}{m}=\frac{y-y_{0}}{v}=\frac{z-z_{0}}{p}$$

where $\left(x_{0},y_{0},z_{0}\right)$ represents the point that lies on the line; *m*, *v*, and *p* represent the vector parameters of the line in the X-axis, Y-axis, and Z-axis directions, respectively.

Assuming the tooth profile point cloud data of the gear under the actual workpiece coordinate system as *P_w_*_1_ = (*x_w_*_1_,*y_w_*_1_,*z_w_*_1_)*^T^* and the tooth profile point cloud data under the ideal workpiece coordinate system as *P_w_* = (*x_w_*,*y_w_*,*z_w_*)*^T^*, based on the coordinate transformation model of the measurement system and the positioning error model of the gear, the data transformation from the ideal workpiece coordinate system to the actual workpiece coordinate system can be expressed as:

(11) $$\left(\begin{array}{c} P_{w1}\\ 1 \end{array}\right)=\left(\begin{array}{cc} A_{w}^{w1} & D_{w}^{w1}\\ 0 & 1 \end{array}\right)\cdot \left(\begin{array}{c} P_{w}\\ 1 \end{array}\right)$$

$A_{w}^{w1}$, $D_{w}^{w1}$ can be expressed as:

(12) $$\left\{\begin{array}{l} A_{w}^{w1}=A_{x}\cdot A_{y}\\ D_{w}^{w1}={\left(-x_{w1},-y_{w1},-z_{w1}\right)}^{T} \end{array}\right.$$

(13) $$A_{x}=\left(\begin{array}{ccc} 1 & 0 & 0\\ 0 & \cos\alpha_{x} & -\sin\alpha_{x}\\ 0 & \sin\alpha_{x} & \cos\alpha_{x} \end{array}\right)$$

(14) $$A_{y}=\left(\begin{array}{ccc} \cos\beta_{y} & 0 & \sin\beta_{y}\\ 0 & 1 & 0\\ -\sin\beta_{y} & 0 & \cos\beta_{y} \end{array}\right)$$

where $\alpha_{x}$ represents the rotation angle of the ideal workpiece coordinate system around the x-axis, $\alpha_{x}=\arccos(p/\sqrt{n^{2}+p^{2}})$, $\beta_{y}$ represents the rotation angle of the ideal workpiece coordinate system around the y-axis, and $\beta_{y}=\arccos(p/\sqrt{m^{2}+p^{2}})$, $\left(x_{w1},y_{w1},z_{w1}\right)$ represents the coordinate value of the origin of the actual workpiece coordinate system in ideal workpiece coordinate system.

Based on the coordinate transformation matrix and the positioning error correction matrix, the tooth profile point cloud data can be obtained with high precision in the actual workpiece coordinate system to achieve a high-precision evaluation of gears.

## 3. Error Model of Small-Modulus Gears

**Figure 7:**
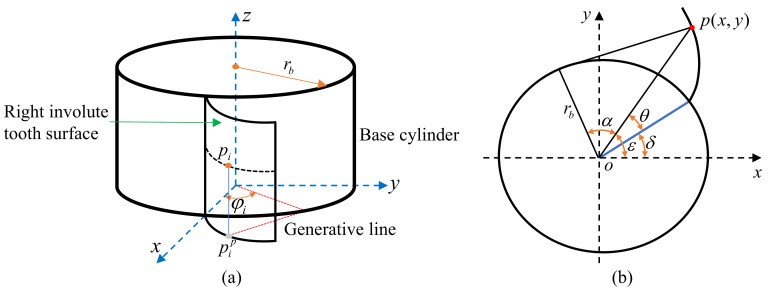
Model: (**a**) 3D model of small-modulus gear; (**b**) Single tooth profile section.

Small-modulus gears are essential transmission components. Accurate error calculation models can be used to analyze and characterize the precision level of small-modulus gears to ensure quality requirements for gear products. The most commonly used tooth profile for small-modulus gears is the involute profile, which is formed when a generating line tangent to the base circle does pure scrolling. Based on the geometric parameters of the spatial tooth flank, the spatial right tooth flank, and the single tooth profile section model of the small-modulus gears are established, as shown in Figure 7. The purpose is to analyze the calculation method for the gear error.

As shown in Figure 7a, a spatial cartesian coordinate system is established based on the center point of the lower surface of the gear. Set the starting point of the involute of the lower surface of the right tooth flank of a certain tooth profile to be on the X-axis. Set up point $p_{i}$ as any point on the spatial involute tooth flank, with $p_{i}^{p}$ its projection on the XY-plane and lying on the involute of the lower surface of the right tooth profile. Based on the standard involute equation for gears, the point $p_{i}$ on the right tooth face of the tooth profile in the current space can be expressed as:

(15) $$\left\{\begin{array}{l} x_{R}(p_{i})=r_{b}(\cos\varphi_{Ri}+\varphi_{Ri}\cdot \sin\varphi_{Ri})\\ y_{R}(p_{i})=r_{b}(\sin\varphi_{Ri}-\varphi_{Ri}\cdot \cos\varphi_{Ri})\\ z_{R}(p_{i})=z_{i} \end{array}\right.$$

where *R* represents the right flank of a small-modulus gear.

As shown in Figure 7b, the point p is any point on the involute of a single tooth profile section, and its coordinate is (x,y). α is the pressure angle at point *p*; θ is the exhibition angle at point *p*; ε is the angle between the line that connects point *p* and the center of the base circle and the X-axis; δ is the angle of the starting point of the involute. Based on the single cross-section standard involute theoretical equation, θ=tanα−α, the expression of the starting angle of any involute can be expressed as:

(16) $$\delta =\arctan(\frac{y}{x})+\arctan(\frac{\sqrt{x^{2}+y^{2}-r_{b}^{2}}}{r_{b}})-\frac{\sqrt{x^{2}+y^{2}-r_{b}^{2}}}{r_{b}}$$

The theoretical Equation (16) is used to extract the profile deviation of the small-modulus gears by the involute fitting method, which can ensure measurement precision.

### 3.1. Model of Profile Deviation

**Figure 8:**
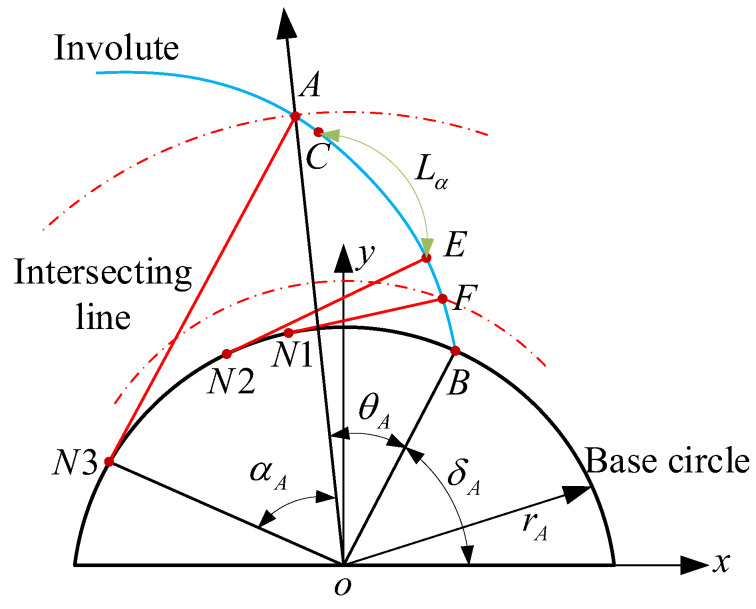
Profile evaluation length.

Profile deviation is defined in the gear end plane, which is caused by machining errors in gear processing, resulting in discrepancies between the actual and theoretical profiles. The size of the profile deviation must be controlled to guarantee the quality and performance of the gear. As shown in Figure 8, profile deviation is calculated within the profile evaluation length; the profile evaluation length $L_{\alpha }$ is 92% of the effective length $L_{AE}$.

**Figure 9:**
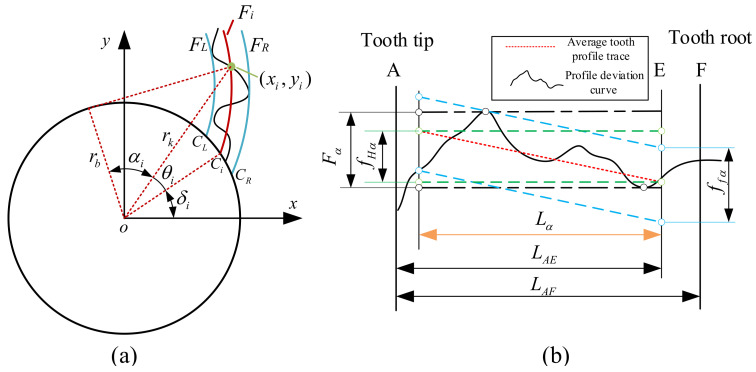
Model: (**a**) Extraction model of profile deviation (**b**) Curve of profile deviation.

In order to obtain the profile deviation, an extraction model of profile deviation is established based on the involute fitting method, as shown in Figure 9a. $F_{i}$ represents the fitted involute at the tooth profile measurement points $\left(x_{i},y_{i}\right)$, $F_{L}$ represents the fitted left limit involute, and $F_{R}$ represents the fitted right limit involute. Based on Equation (17), the starting point $C_{i}$ of the fitted involute at any measuring point in the profile evaluation length can be obtained, which is located on the base circle. Suppose that the starting point of the theoretical involute is *C*, the rotation angle when *C* rotates around the origin *o* and passes through $C_{i}$ in turn is $\Delta \delta_{i}$, which can be expressed as:

(17) $$\Delta \delta_{i}=\arccos\frac{x_{i}\cdot \cos\delta +y_{i}\cdot \sin\delta }{r_{b}}$$

where $r_{b}$ represents the radius of the base circle, and δ represents the starting angle of theoretical involute. The profile deviation of any measuring point can be expressed as $F_{\alpha i}=r_{b}\cdot \Delta \delta_{i}$, $F_{\alpha L}=r_{b}\cdot \Delta \delta_{i}$ is the maximum value of $F_{\alpha i}$, and $F_{\alpha R}=r_{b}\cdot \Delta \delta_{R}$ is the minimum value of $F_{\alpha i}$. It is assumed that, within the profile evaluation length $L_{\alpha }$, the expansion length of the measurement points are $L_{i}$. Taking $L_{i}$ as the horizontal coordinates and $F_{\alpha i}$ as the vertical coordinate, the profile deviation curve can be plotted based on the profile deviation point dataset $\left(L_{i},F_{\alpha i}\right)$, as shown in Figure 9b.

The evaluation index of profile deviation consists of Profile Total Deviation $F_{\alpha }$, Profile Form Deviation $f_{f\alpha }$ and Profile Slope Deviation $f_{H\alpha }$. In the range of profile evaluation length, the $F_{\alpha }$ represents the absolute value of the difference between the maximum and minimum deviation values, and the $f_{f\alpha }$ and $f_{H\alpha }$ are determined by least squares fitting of the average tooth profile trace. The profile form deviation can be expressed as:

(18) $$f_{f\alpha }=\left|\max(\Delta F_{\alpha i}-y_{i})-\min(\Delta F_{\alpha i}-y_{i})\right|$$

where $\Delta F_{\alpha i}$ represents the profile deviation at a point; $y_{i}$ represents the fitted average tooth profile deviation value.

### 3.2. Model of Pitch Deviation

Pitch deviation is defined as the difference between the actual pitch and the theoretical pitch on the reference circle, which is one of the essential indexes for gear precision and performance evaluation. It has an important influence on the efficiency, noise and vibration, transmission precision, and service life of gears. The single theoretical pitch can be expressed as:

(19) $$p=r\cdot \theta =r\cdot \frac{2\pi }{z}$$

where *r* represents the radius of the reference circle, θ represents the theoretical radian of a single pitch, and *z* represents the number of gear teeth.

The evaluation index of pitch deviation consists of Individual Single Pitch Deviation $f_{pi}$, Single Pitch Deviation $f_{p}$, Individual Cumulative Pitch Deviation $F_{pi}$ and Total Cumulative Pitch Deviation $F_{p}$ based on ISO 1328-1:2013 [24]. Assuming that the intersection line between the reference cylinder and the involute tooth flank is the theoretical reference line, therefore the point at which the theoretical tooth pitch is calculated is obtained by the intersection of the reference circle of each end section of the gear with the theoretical reference line.

**Figure 10:**
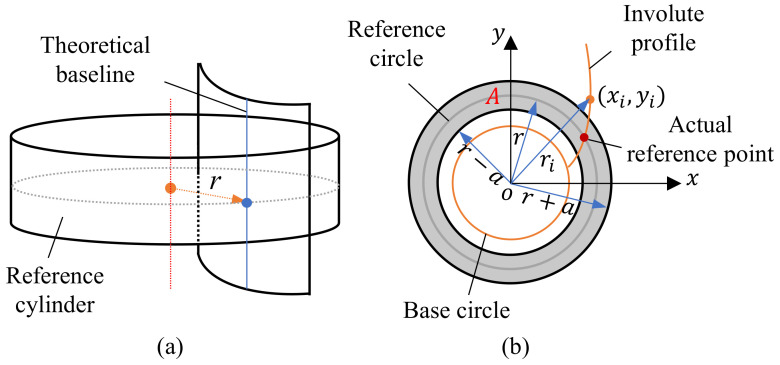
Model: (**a**) Theoretical model of three dimensions (**b**) Data Extraction Model of region A.

The pitch deviation of a single section is calculated on the gear reference circle, as shown in Figure 10a. Setting the threshold *a* with [−*a*, *a*] as the benchmark, as shown in Figure 10b, the measurement data points of region *A* are extracted by Equation (20) for tooth profile fitting. The actual reference point is obtained by calculating the intersection point of the fitted tooth profile and the reference circle to obtain the actual pitch value. In this way, the number and distribution of discrete points of tooth profile near the indexing circle can be balanced, and the accuracy of the actual reference point can be ensured.

(20) $$p_{A}(x_{i},y_{i})=\left\{(x_{i},y_{i})\left|\left|r_{i}-r\right|\leq a\right.\right\}$$

where *r* represents the radius of the reference circle, and $r_{i}$ represent the distance from the measuring point to the gear shaft. In this paper, the calculation of pitch deviation is obtained by setting the initial profile and calculating the individual single pitch deviation value in a counterclockwise direction on the reference circle.

## 4. Gear Measurement Experiment

The measurement experiment is carried out using a small-modulus cylindrical spur gear of 0.1 mm modulus 6-grade precision, and the sub-item errors of the gear are decomposed to verify the method. The details of the experimental setup are introduced in this section and the detailed parameters of the gear used in the experiment are shown in Table 3.

**Figure 11:**
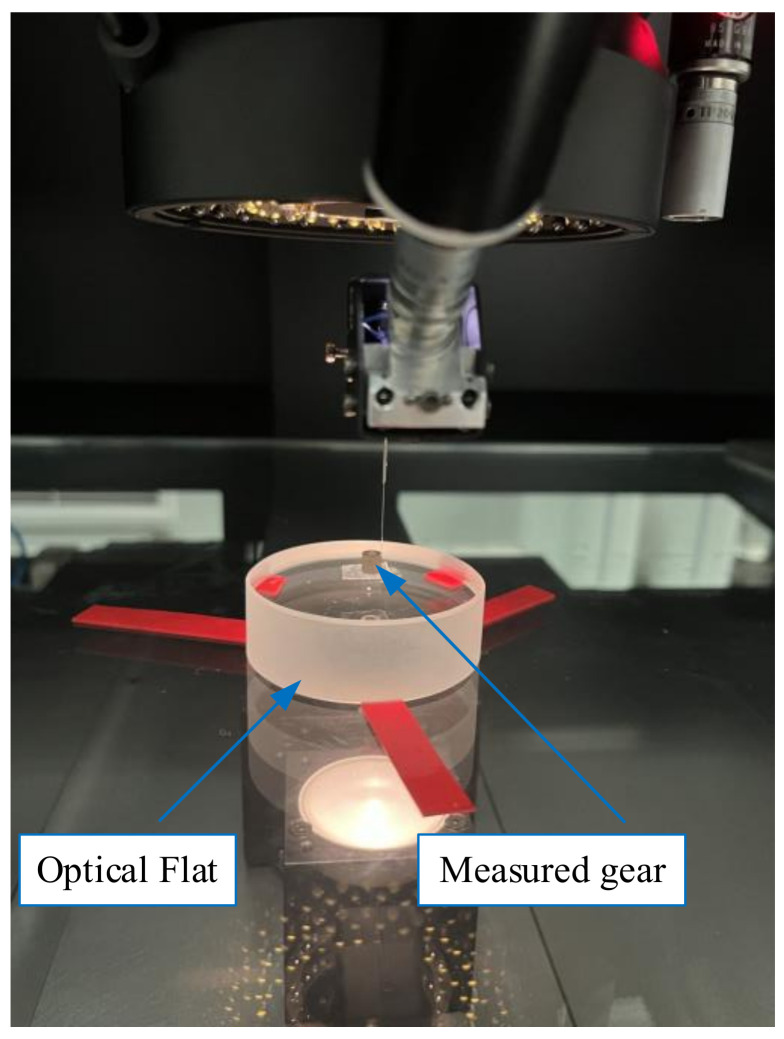
Optical fiber sensor.

**Figure 12:**
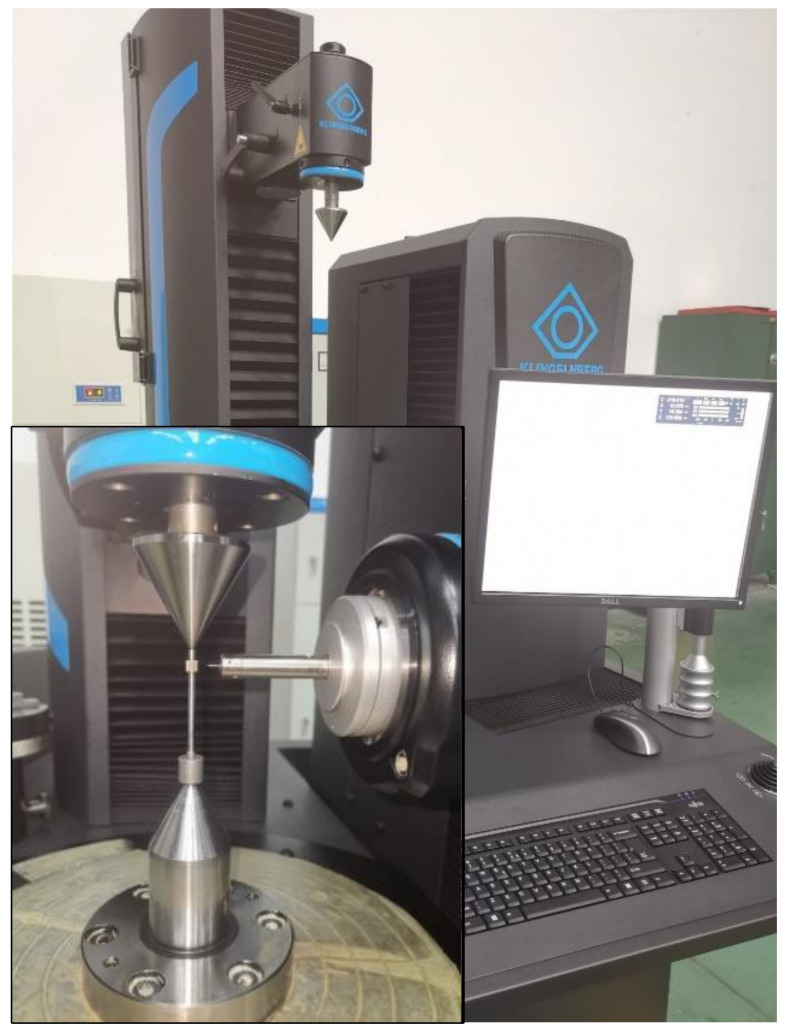
P26 gear measuring center.

As shown in Figure 11, the small-modulus gear measurement experiment is conducted using the fiber optic sensor to obtain the three-dimensional point cloud data of the gear. This method can only obtain data for a single tooth profile section during one measurement. To analyze gear errors on a three-dimensional scale, a certain step size is set to measure multiple tooth profile cross-section data in the width direction of the tooth. The measured gear is fixed on the operating board by an optical flat, which can eliminate some positioning errors and ensure the accuracy of the original data. Similarly, the comparative experiment is conducted using P26, as shown in Figure 12.

According to the characteristics of the small-modulus gear used in this study, it is necessary to use the discrete point data of multiple involute tooth profiles to reverse the geometric center of the gear, so as to avoid the influence of the insufficient number of discrete points of the tooth profile in the single involute area, and the layout distribution of the multiple involutes in the circumferential direction is uniform to ensure the accuracy of the reverse result of the gear center. Based on three tooth profile measurement sections, the inverse results of the gear geometric center are shown in Table 4.

**Figure 13:**
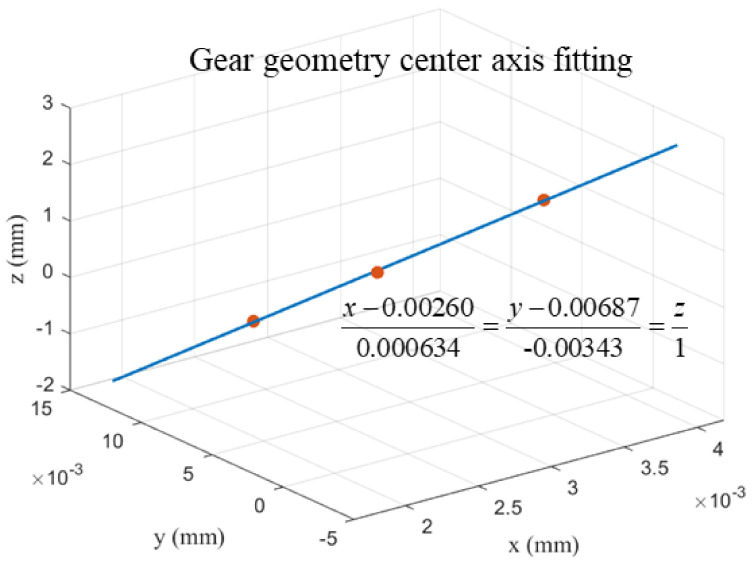
Gear center geometric axis fitting.

Based on the geometric center value of the gear in the above table, a space straight line linear fitting is carried out, and the result is shown in Figure 13. Take this spatial straight line as the *z_w_*_1_ axis of the actual workpiece coordinate system of the gear and choose any point on this straight line as the origin *o_w_*_1_ of the coordinate system, so as to establish the actual workpiece coordinate system *o_w_*_1_-*x_w_*_1_*y_w_*_1_*z_w_*_1_. According to the fitted gear geometric axis equation, the ideal workpiece coordinate system needs the rotation angle *α_x_* = 0.20° (counterclockwise direction) around the x-axis and the rotation angle *β_y_* = −0.036° (clockwise direction) around the y-axis to obtain the tooth profile point cloud data in the actual workpiece coordinate system.

Based on the mathematical motion model and coordinate transformation model in Section 2.1 and Section 2.2, the position relationships between each coordinate system are established within the measurement system. The position parameters between coordinate systems are then determined by the structural parameters of the measurement platform, resulting in the creation of the coordinate transformation matrix. By combining the measurement principle and considering the positioning error correction method, the point cloud data of the small-modulus gear tooth profile in the actual workpiece coordinate system can be extracted.

## 5. Measurement Data Processing and Discussion

During measurement, the CCD camera acquires an image of the gear, and then the measurement software is used to set the initial position for tooth profile measurement, the measurement direction, and the step size in the tooth width direction.

**Figure 14:**
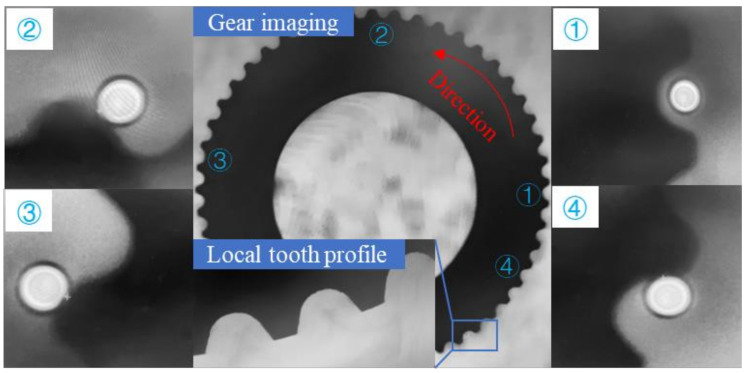
Small-modulus gear measurement imaging.

**Figure 15:**
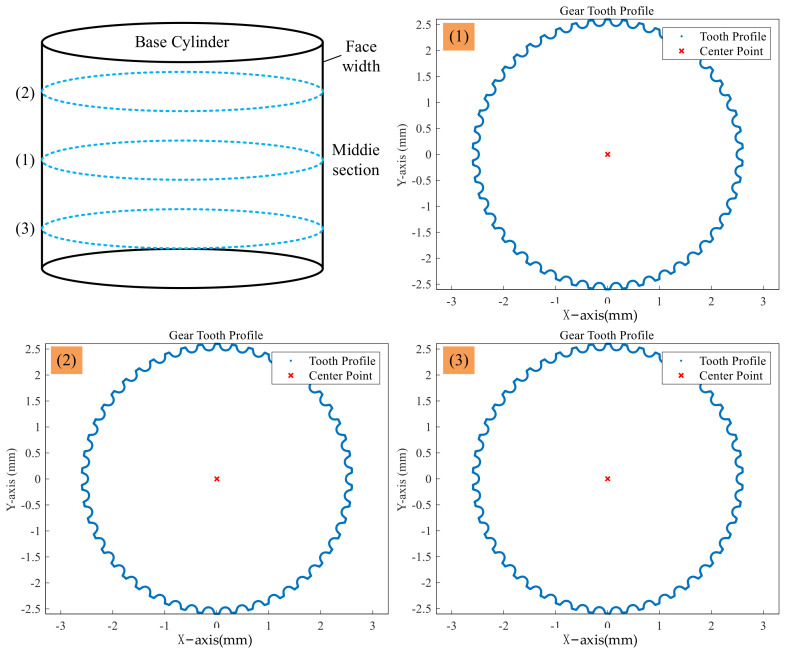
The point cloud of single section tooth profile.

As shown in Figure 14, the starting point and direction set during the measurement and the four position states of the fiber optic sensor are given. The point cloud data of multiple tooth profile sections are collected in the actual workpiece coordinate system based on the coordinate transformation matrix, as shown in Figure 15. The measurement area covers the entire tooth profile curve of a single gear tooth profile, with approximately 5500 measurement points.

The output of the fiber optic sensing device is a two-dimensional single cross-section, and the median filter is used for filtering and noise reduction to pre-process the data after acquisition. However, the filtered measurement data cannot be used to calculate the sub-item errors, and it is necessary to determine further the area where the data are located. Taking the difference between the abscissa of the latter point of the measured data and the abscissa of the former point as the step length, the forward difference method is used to segment the data.

(21) $$\Delta y_{i}=\frac{y_{i+1}-y_{i}}{x_{i+1}-x_{i}}$$

(22) $$\Delta^{2}y_{i}=\frac{\Delta x_{i+1,i}\cdot y_{i+2}-\Delta x_{i+2,i}\cdot y_{i+1}+(\Delta x_{i+2,i}-\Delta x_{i+1,i})\cdot y_{i}}{\Delta x_{i+1,i}\cdot \Delta x_{i+2,i}\cdot (\Delta x_{i+2,i}-\Delta x_{i+1,i})}$$

**Figure 16:**
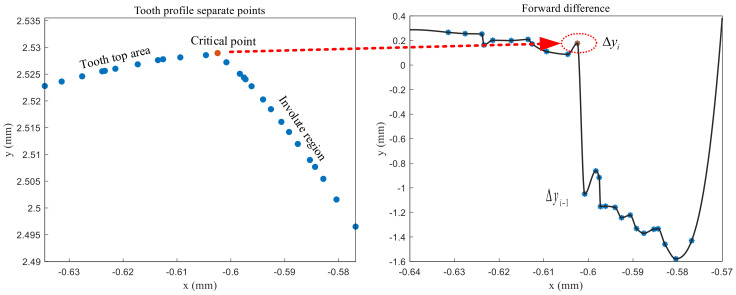
The critical points of the addendum region and the involute region.

**Figure 17:**
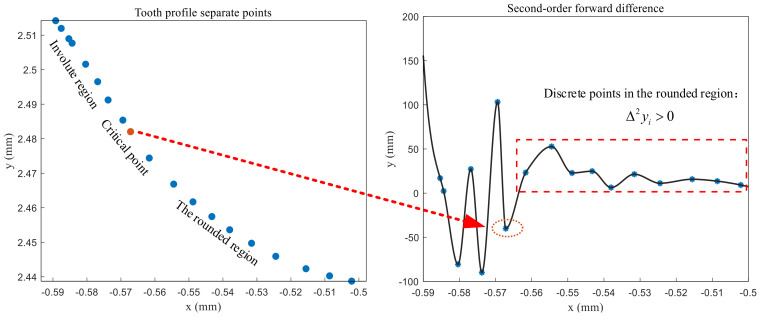
The critical points of the involute region and the rounded region.

By judging the changes of $\Delta y_{i}$ and $\Delta^{2}y_{i}$, the critical points of the involute region in the addendum circle region and the fillet region can be found, as shown in Figure 16 and Figure 17.

### 5.1. Profile Deviation

**Figure 18:**
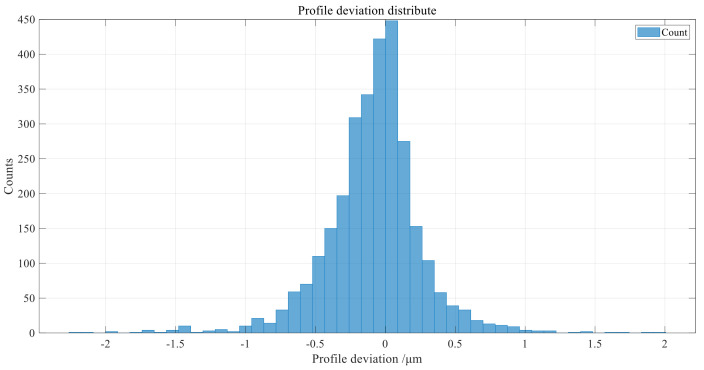
Point-by-point tooth profile deviation distribution.

Based on the extracted tooth profile measurement points of the left and right tooth profile involute regions, according to the small-modulus gear tooth profile deviation model proposed in Section 3.1, the tooth profile data points within the tooth profile evaluation length of each involute tooth profile region are extracted to calculate the tooth profile deviation data. The tooth profile deviation distribution of all tooth profile data points is shown in Figure 18, which observes the difference between the actual tooth profile and the theoretical tooth profile of the gear as a whole and reflects the quality level of gear processing.

**Figure 19:**
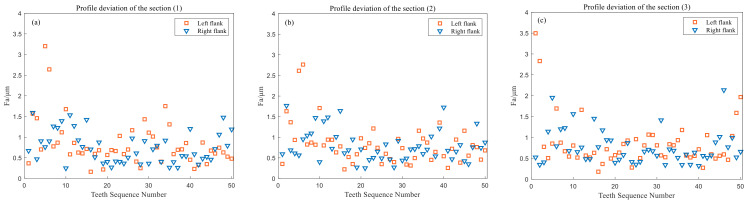
Measurement results of *F_αi_*. (**a**): Section (1); (**b**): Section (2); (**c**): Section (3).

The measurement results of the tooth profile deviation of the left and right tooth profiles of each section are shown in Figure 19.

The measurement results of the total deviation of the three cross-section profiles are shown in Table 5.

**Figure 20:**
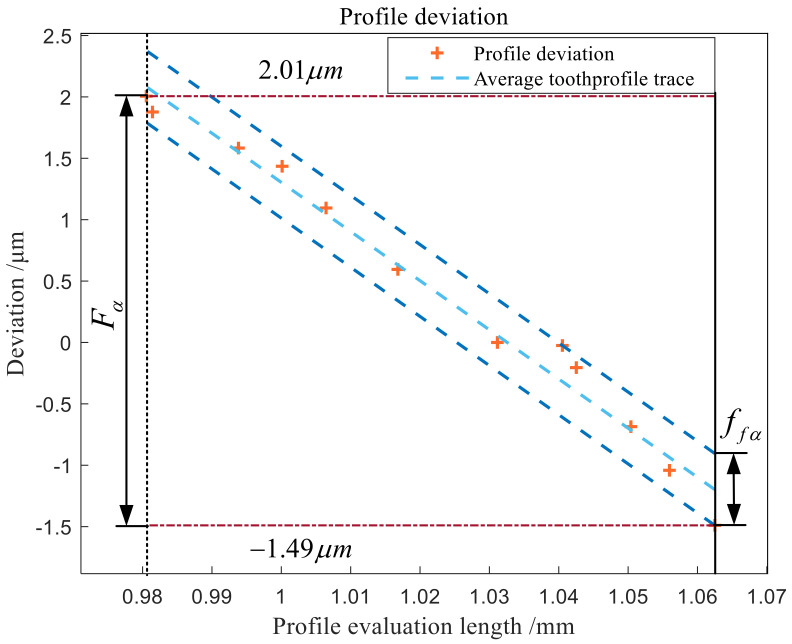
Profile deviation data curve.

By evaluating the gear on the three-dimensional scale, the profile total deviation extracted is 3.50 μm, and the corresponding precision grade is 4, which corresponds to the left tooth profile of the first tooth profile of Section 3. The tooth profile data points of the involute in the range of tooth profile measurement are expanded, and the tooth profile deviation data curve is drawn, as shown in Figure 20. The tooth profile shape deviation is 0.59 μm, and the tooth profile inclination deviation is −3.45 μm.

**Figure 21:**
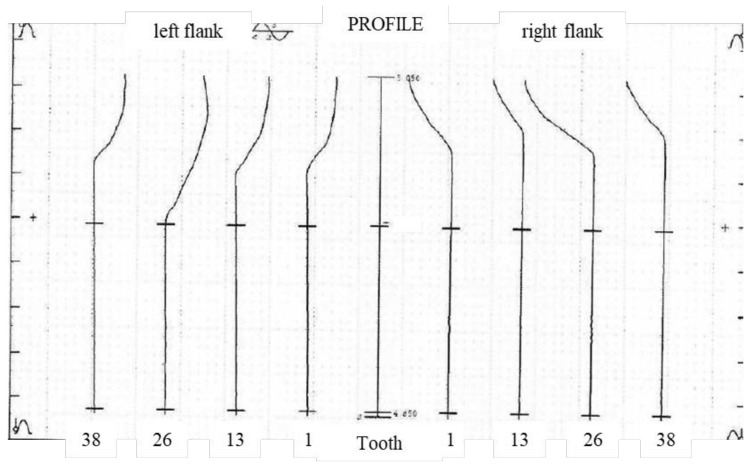
Measurement results of P26.

As shown in Figure 21, the profile total deviation $F_{\alpha }$ measurement result of P26 Gear Measuring Center is 0.8 μm. The difference between the two instruments in the measurement of profile total deviation is about 2–3 μm.

### 5.2. Pitch Deviation

**Figure 22:**
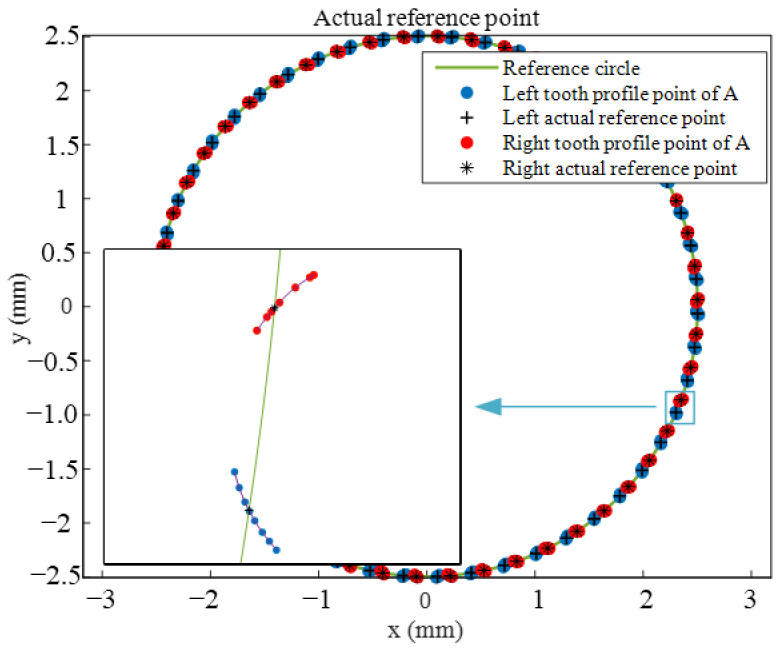
Actual reference point of actual pitch.

Based on the pitch deviation calculation model proposed in Section 3.2 and the constraint conditions, the tooth profile data points near the reference circle are extracted. Then, based on the cubic spline interpolation algorithm, the tooth profile is fitted to obtain the actual reference point of the measured tooth profile at the reference circle position, as shown in Figure 22.

**Figure 23:**
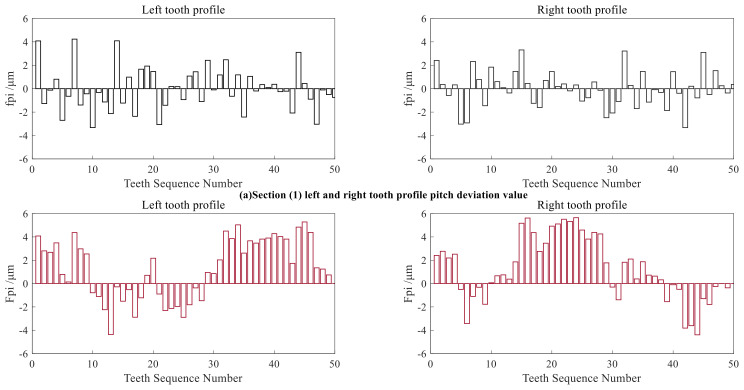

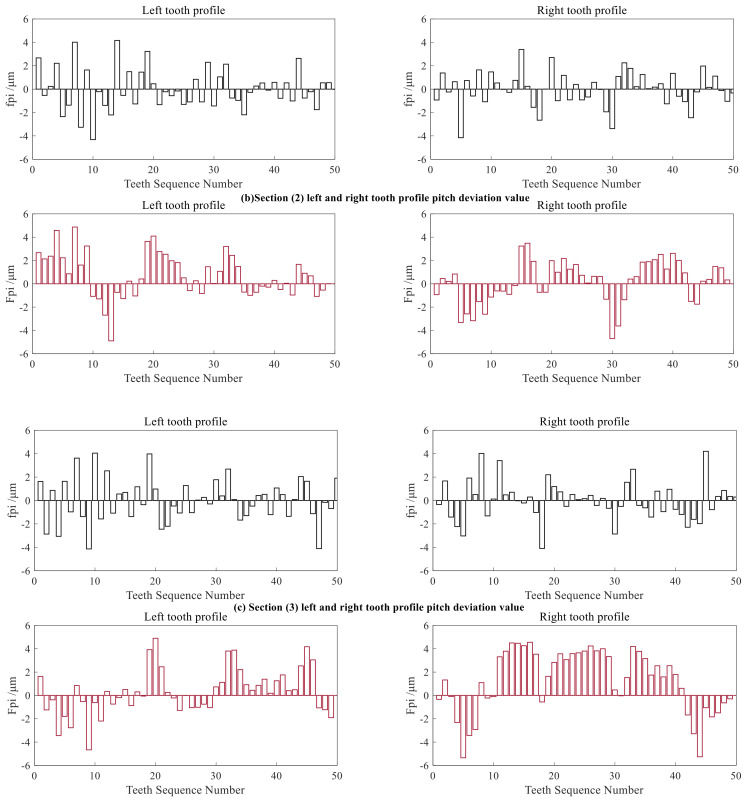
Measurement results of *f_pi_* and *F_pi_*. (**a**): Section (1); (**b**): Section (2); (**c**): Section (3).

By calculating the arc lengths between the actual datum points and comparing them with the arc lengths between the theoretical datum points, the single pitch deviation and the total cumulative pitch deviation of the small-modulus gear can be obtained. The measurement results of pitch deviation of all left and right tooth profiles of each section are shown in Figure 23. The measurement results of the three tooth profile sections are shown in Table 6.

The measurement results show that the single pitch deviation $f_{p}$ of the gear is 4.32 μm, the corresponding precision grade is 6, the total cumulative pitch deviation $F_{p}$ is 10.04 μm, and the corresponding precision grade is 5.

**Figure 24:**
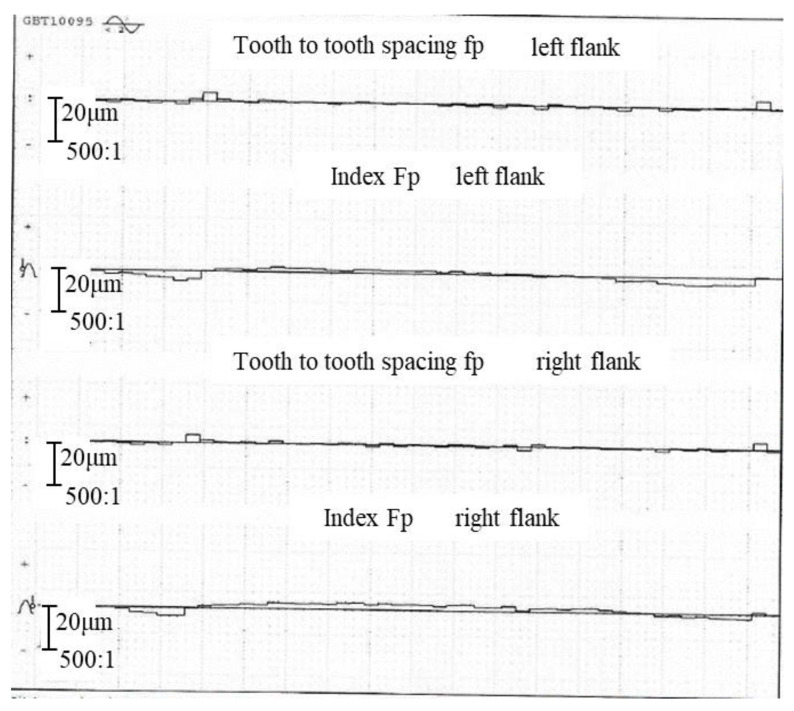
Measurement results of P26.

The P26 gear measuring center is used to measure the gear, and the pitch error curve is shown in Figure 24. According to the measurement results, the single pitch deviation $f_{p}$ is 4.0 μm, the corresponding precision grade is 6, the total cumulative pitch deviation $F_{p}$ is 7.2 μm, and the corresponding precision grade is 5. By comparing the results of pitch deviation, the proposed deviation extraction model can accurately measure pitch deviation. When measuring small-modulus gears, the difference between the two instruments in the measurement of single pitch deviation is relatively small with good consistency, and the difference in the measurement of total cumulative pitch deviation is about 2–3 μm.

The possible reasons for the differences are as follows: (1) There is still a certain positioning error after the positioning error correction of the gear, which affects the acquisition accuracy of the tooth profile data; (2) Different threshold settings will affect the accuracy of tooth profile fitting when extracting the three-dimensional point cloud data of tooth profile in the area *A* near the dividing circle.

### 5.3. Discussion

The measurement experiment is conducted using a small-modulus gear of 0.1 mm modulus 6-grade precision, and the sub-item errors of the gear are extracted. By measuring multiple tooth profile sections, the precision of the gear is evaluated at the three-dimensional scale. The measurement result shows the profile total deviation $F_{\alpha }$ is 3.50 μm, the single pitch deviation $f_{p}$ is 4.32 μm, and the total cumulative pitch deviation $F_{p}$ is 10.04 μm. The measurement result is compared with the measurement result of the P26 gear measurement center, which verifies the correctness of the proposed method. The method has a certain practicability for the measurement of small-modulus gears and can be used for high-precision measurement of small-modulus gears with 0.1mm modulus 6-grade precision.

## 6. Conclusions

In this paper, a small-modulus gear ‘contact-optical’ compound measurement system based on coordinate measuring machine and optical fiber sensing is built. By studying the 3D model and mathematical motion relationship of the measurement system, the spatial coordinate transformation model between the CCD telecentric camera coordinate system, the machine coordinate system and the workpiece coordinate system is established. To improve the measurement accuracy of small-modulus gears, the formation mechanism of gear positioning error is analyzed, and a spatial coordinate transformation matrix optimization method considering gear positioning error is proposed. To construct the actual workpiece coordinate system of the gear, the reverse method of the geometric center of the single tooth profile section of the gear is studied. Based on the established small-modulus gear positioning error model, the mapping relationship between the ideal workpiece coordinate system and the actual workpiece coordinate system is determined, and the collected tooth profile measurement points are transformed from the optical fiber sensing coordinate system to the actual workpiece coordinate system. Moreover, the extraction model and algorithm of partial errors, such as gear pitch deviation and tooth profile deviation, are studied. The method proposed provides a new measurement scheme for small-size gears with small-modulus. Accurate measurement results can guide the manufacturing of micro gears.

## Figures and Tables

**Table 1:** Geometric and accuracy parameters of CMM.

Parameter	Value
Measurement range (X-axis)	400 mm
Measurement range (Y-axis)	400 mm
Measurement range (Z-axis)	400 mm
Uniaxial accuracy	(0.5 + L/900) μm
XY plane accuracy	(1.0 + L/400) μm
Volumetric accuracy	(1.5 + L/300) μm
Temperature	20 ± 0.5 K and 0.25 K/h

**Table 2:** Parameters and accuracy of fiber optic sensing devices.

Parameter	Value
Probe-diameter	25 μm
Contact force	≤1 μN
Point detection error	0.3 μm
Scanning probing error	0.3 μm

**Table 3:** Experimental gear parameters.

Parameter	Value
Normal gear modulus $m_{n}$	0.1 mm
Number of teeth *z*	50
Gear width *b*	5 mm
Normal pressure angle $\alpha_{n}$	20°
Pitch diameter *d*	5 mm
Precision grade	6

**Table 4:** Inverse results of gear geometric center.

Number	Gear Geometric Center Coordinates(/mm)
1	(0.0036, 0.0017, 1.54)
2	(0.0028, 0.0052, 0.41)
3	(0.0023, 0.0088, −0.52)

**Table 5:** Measurement results of profile total deviation *Fα.*

Section	$\mathrm{Profile}\;\mathrm{Total}\;\mathrm{Deviation}\;F_{\alpha }$
(1)	3.20 μm
(2)	2.76 μm
(3)	3.50 μm

**Table 6:** Measurement results of pitch deviation.

	$\mathrm{Sin}\mathrm{gle}\;\mathrm{Pitch}\;\mathrm{Deviation}\;f_{p}$	$\mathrm{Total}\;\mathrm{Cumulative}\;\mathrm{Pitch}\;\mathrm{Deviation}\;F_{p}$
(1)	4.23 μm	10.04 μm
(2)	4.32 μm	9.77 μm
(3)	4.20 μm	9.90 μm

## Data Availability

The authors attest that all data for this study are included in the paper.

## Nomenclature

$r_{b}$	Base radius [mm]
d	Reference circle diameter [mm]
b	Gear width [mm]
$m_{n}$	Normal modulus [mm]
Z	Number of teeth
$F_{\alpha }$	Profile Total Deviation [μm]
$f_{f\alpha }$	Profile Form Deviation [μm]
$f_{H\alpha }$	Profile Slope Deviation [μm]
$f_{pi}$	Individual Single Pitch Deviation [μm]
$f_{p}$	Single Pitch Deviation [μm]
$F_{pi}$	Individual Cumulative Pitch Deviation [μm]
$F_{p}$	Total Cumulative Pitch Deviation [μm]
$L_{\alpha }$	Profile evaluation length [mm]
$L_{AE}$	Profile effective length [mm]
$L_{AF}$	Profile length [mm]
φ	Profile expansion angle [°]
$x_{i}$	Measured value on profile x [mm]
$y_{i}$	Measured value y [mm]
$o_{w}-x_{w}y_{w}z_{w}$	Workpiece coordinate system
$o_{m}-x_{m}y_{m}z_{m}$	Machine coordinate system
$o_{x}-x_{x}y_{x}z_{x}$	X-axis fixed coordinate system
$o_{y}-x_{y}y_{y}z_{y}$	Y-axis fixed coordinate system
$o_{z}-x_{z}y_{z}z_{z}$	Z-axis fixed coordinate system
$o_{c}-x_{c}y_{c}z_{c}$	CCD telecentric camera coordinate system
